# Efficacy of Amitraz plus Metaflumizone for the treatment of canine demodicosis associated with *Malassezia pachydermatis*

**DOI:** 10.1186/1756-3305-2-13

**Published:** 2009-03-05

**Authors:** Viviana D Tarallo, Riccardo P Lia, Mariateresa Sasanelli, Claudia Cafarchia, Domenico Otranto

**Affiliations:** 1 Department of Veterinary Public Health and Animal Sciences Faculty of Veterinary Medicine, University of Bari, 70010, Valenzano, Bari, Italy

## Abstract

This case reports the efficacy of metaflumizone plus amitraz spot-on formulation (ProMeris Duo^®^; Fort Dodge) against generalized demodectic mange. A two year-old male dog presented at clinical examination with poor general condition, diffused alopecia, crusted lesions, pruritus, skin scales and pustules. *Demodex *mites, *Malassezia pachydermatis *yeasts and bacteria were diagnosed. The dog was treated with cephalexin and topically with metaflumizone plus amitraz spot on formulation at two weeks intervals until two consecutive skin scrapings resulted negative for mites. The number of adult mites statistically decreased at follow-up with a reduction of approximately 42 and 94% at +14 and +28 days post treatment (p.t.) respectively. Nymphs and larvae could not be detected from +28 day p.t. while eggs were no longer present +42 day p.t. The dog was negative for both bacteria and *M. pachydermatis *at 14 days p.t., coinciding with improved general clinical conditions, recovering skin lesions and no further signs of pruritus. These results show that metaflumizone plus amitraz associated with the antibiotic therapy is highly effective for treating generalized demodectic mange and could also be effective toward controlling *M. pachydermatis *opportunistic infections.

## Findings

*Demodex canis *(Acari, Demodicidae), a common component of canine skin, can cause skin disease in immune-depressed subjects with symptoms including localized or diffuse alopecia, erythema, comedones associated with papular and pustular dermatitis [1]. Indeed, a high number of *Demodex *spp. mites within the follicles and sebaceous glands causes canine demodicosis [1], which is often associated with folliculitis and furunculosis due to secondary bacterial and/or mycotic infections [2]. Among the latter are *Malassezia *spp. lipophylic yeasts that are part of the normal cutaneous microflora of most warm-blooded animals. These may suddenly act as opportunistic pathogens causing dermatitis or otitis in dogs [3]. In dogs, a significant proliferation of *Malassezia pachydermatis *is likely a preliminary step toward dermatitis and/or otitis [4,5], its overgrowth being caused by changes in the cutaneous microenvironment and/or alterations of host defence mechanisms [3]. Under the above circumstances both *Demodex *spp. infection and *Malassezia *overgrowth may be responsible for severe skin lesions in dogs [2,3].

The clinical presentation of demodectic mange depends on the age of the infected animals [6]. Young dogs (< 2 years old) can be affected by localized or generalized forms of demodicosis based on the extension and localization of the lesions [1]. Indeed, the presence of more than five localized lesions on the body, or of two or more feet affected by parasitic lesions, are indicative of generalized infection [6]. In adult dogs (i.e. > 4 years of age) demodectic mange may occur as a generalized form [6]. It has been proposed that the critical factors for the appearance of clinical signs of demodectic mange are genetic predisposition (e.g. breed) and/or immunosuppressive conditions (e.g. neoplasia, diabetes, hyperadrenocorticism and metabolic disease) [1].

The diagnosis of mange is usually based on the detection of mites in skin scrapings both in presence or absence of lesions. Treatment is based on chemical control of the mites and supportive therapy for opportunistic *Malassezia *or bacterial infection [2]. Juvenile-onset localized demodectic mange resolves without treatment in 90% of cases, whereas in some patients the administration of topical or oral antibiotics is needed in order to control the secondary bacterial skin infections [6]. Conversely, generalized demodectic mange is a serious condition requiring a prolonged pharmacological treatment, which is often considered difficult and potentially life-threatening [7]. Because the life cycle of the mite extends over a period of 18–24 days [8] and due to the difficulties experienced with the treatment of generalized demodicosis, multiple approaches are advised.

Amitraz is the only molecule approved by the Food and Drug Administration (FDA) for the treatment of canine demodicosis [6]. This molecule belongs to the formamidine family and acts by inhibiting monoamine oxidase and prostaglandin synthesis and by stimulating the alfa 2 – adrenergic receptors of the arthropod nervous system. Treatment protocols vary according to the extent and severity of lesions. Whole-body rinses to topical treatment of localized lesions (e.g. pododemodicosis), at different concentrations and intervals [9,10]. Several side effects (e.g. lethargy, depression, anorexia, vomiting, diarrhoea, hypothermia, ataxia, pruritus, bradycardia, hyperglycemia) were reported in some breeds (e.g. collie) and, more rarely, in owners handling the drug [6]. Alternatively macrocyclic lactones (i.e. avermectins and milbemycines) are also used as pour-on, oral or injectable formulations to treat generalized demodicosis [7,10].

A novel amitraz (499,5 mg) plus metaflumizone (499,5 mg) association in a spot-on formulation (ProMeris Duo^®^; Fort Dodge, Animal Health, Overland Park, KS) has recently been registered for treatment of flea and tick infections in dogs [11,12]. Metaflumizone is an insecticide blocking the passage of sodium through nervous cell membranes in arthropods, preventing the onset of nervous impulses. The association has shown good efficacy in treating generalized canine demodectic mange with a significant reduction of the number of adult mites and eggs at 14 or 28 days post treatment [7]. The aim of the present study was to describe a case report of severe generalized demodectic mange associated to *Malassezia *infection and to evaluate the efficacy and the safety of amitraz (499.5 mg) plus metaflumizone (499.5 mg) spot-on formulation for its treatment.

**Figure 1:**
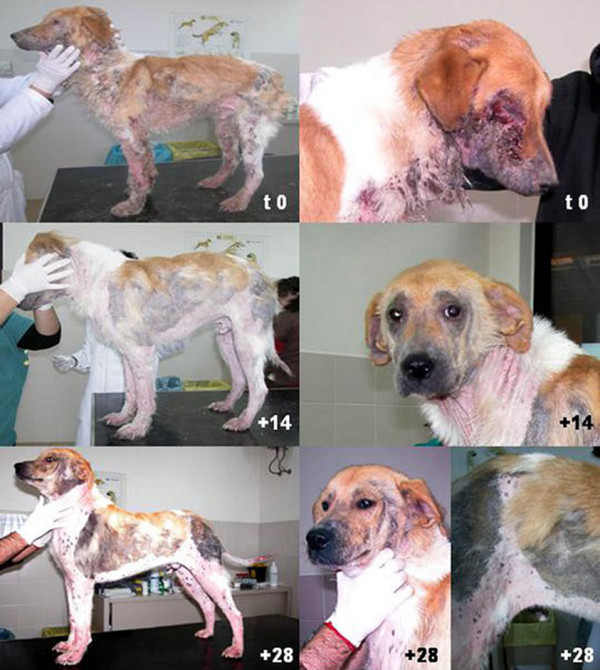
**Patient at day 0 (before treatment) and on day +14 and +28, post treatment**.

In February 2008, a two year-old male mixed-breed dog, weighing 18 kilograms, was referred by the owner to the Parasitological Unit of the Faculty of Veterinary Medicine of Bari (Apulia region, Southern Italy). The animal presented with poor general condition and showed lymph-node enlargement and fever (40°C). The dog exhibited seborrhoea on the whole body surface with extensive areas of alopecia, crusted lesions which were more severe on the legs, neck and face, and pruritus (Figure 1). In particular, the patient showed diffused bleeding wounds around the eyes and serious traumatic corneal lesions. Based on the animal's history and physical examination, the differential diagnosis included ectoparasitic infections, leishmaniosis, neoplasia, mycobacteriosis, dermatophytosis and combined anaerobic and aerobic bacterial infections. Anti-*Leishmania *antibodies were not detected by Indirect Fluorscence Antibody Test (IFAT). The haemogram revealed neutrophilic leucocytosis while the serum chemistry panel showed no abnormal findings except for hyperproteinemia and hyperglobulinemia.

*Demodex *spp. mite infestation was diagnosed by deep skin samples collected from three anatomical sites (i.e. anterior left leg, thorax and tight). Each scraping was performed on a skin area of approximately 1 square cm. Further samples were taken at day 0 (before treatment) and on days +14, +28, +42, +56, +70 and +84 post treatment (p.t., i.e. until two consecutive skin scrapings were found negative). Samples were cleared in 10% KOH solution, for 15 minutes at room temperature, and observed under the microscope. The number of immature stages (larvae and nymphs), adults and eggs were counted at each scraping and recorded separately. The animal was also clinically evaluated by systematically recording the extent of skin lesions and photographed at each follow-up.

*Malassezia *spp. infection was diagnosed in five anatomical sites (i.e., inguinal, interdigital, external ear canal, perioral, and periorbital areas) using sterile cotton swabs moistened with sterile saline (0.9% NaCl) solution [4,5]. Samples were taken at the same time-points as above. A total of 35 swabs were collected and inoculated on modified Dixon's agar within two hours from collection and Petri dishes were incubated at 32°C for seven days. Because no more than 300 non-confluent colonies of *Malassezia *per plate can be clearly identified by visual inspection, the maximum number of colonies counted per plate was 300. The results were reported as number of colony forming units (CFU) per swab. Four colonies from each positive sample were sub-cultured in modified Dixon's agar for subsequent categorization using the different Tween (i.e. 20, 40, 60, 80) assimilation methods as previously described [13]. The tryptophan and cremophor EL (PeG 35 castor oil-Sigma-Aldrich, Italy) assimilation test and the splitting of esculin were used as additional tests for the differentiation of lipid-dependent yeasts [14,15].

At each follow-up, bacterial infections were evaluated by cytological examination using sterile swabs for sampling. The collected material was smeared on a clean glass slide, heat-fixed and stained with May-Grunwald Giemsa for microscopic examination. Bacterial infection was confirmed if more than 2 cells morphologically identifiable as *cocci *were found in five microscopic fields at 40× magnification [16].

The dog was topically treated with the spot-on formulation containing 499.5 mg of metaflumizone and 499.5 mg of amitraz. The compound was administered on the skin of the animal according to the manufacturer's instructions. The dog was treated at two-week intervals on days 0, +14, +28, +42, +56, +70 and +84. Cephalexin (30 mg/kilograms/12 h for 4 weeks) was also administered to treat the concurrent pyoderma until the bacterial cytological examination tested negative. The differences in mean numbers of *Demodex *spp. mites after treatment with metaflumizone plus amitraz at 14 days intervals were statistically analysed using the T-student test. A value of P = 0.05 was considered statistically significant.

**Figure 2:**
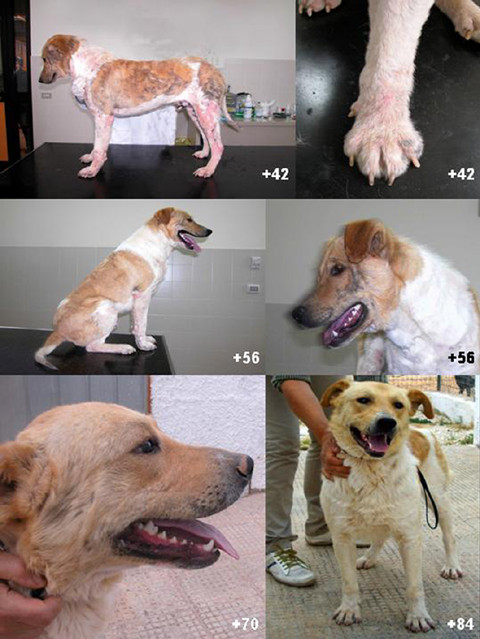
**Patient at day +42, +56, +70 and +84 post treatment**.

The results of parasitological examination from each skin site at each observation time are reported in Additional file 1. At day 0 each sample was positive for different stages of *Demodex canis *(Additional file 1) with the highest parasitic load recorded on the thorax and thighs. The number of adult mites statistically decreased at follow-up with a reduction of approximately 42 and 94% at +14 and +28 days p.t. respectively. Nymphs and larvae could no longer be detected from day +28 p.t., while eggs were no longer observed from day +42 p.t. At the same time, general condition improved and skin lesions resolved, and clinical signs of pruritus disappeared at +14 day p.t. (Figure 1). Lesions due to scratching rapidly resolved over the whole body surface, as well as around the eyes, resulting in hair growth and resolution of alopecic areas. At +70 day p.t. the animal presented in good general condition, including a weight gain of +7 kilos (Figure 2). No adverse reactions to treatment were recorded at any of the follow-ups.

The results of mycological examination from each skin site at each observation time are reported in Table 1. Overall, 52 isolates were collected, identified biochemically as *M. pachydermatis *and isolated from each anatomical site at time +0 and +14 p.t. except for the periorbital area. *M. pachydermatis *was retrieved throughout the entire observation time from the external ear canal with a significant decrease (p < 0.05) in the population size (Table 1). The results of cytological examination revealed the presence of bacterial infection up to +14 day p.t.

**Table 1:** Population size of *Malassezia pachydermatis *expressed as number of colony forming units (CFU) per swab from different anatomical regions and sampling time.

Sampling Sites	0	+14	+28	+42	+56	+70	+84
Inguinal area	78	68	neg	neg	neg	neg	neg
Interdigital area	120	80	neg	neg	neg	neg	neg
External Ear Canal	300	250	45	37	36	24	18
Perioral	32	16	neg	neg	neg	neg	neg
Periorbital	neg	neg	neg	neg	neg	neg	neg

Means (standard deviation)	106 (117.6)	86.8 (99.6)	9 (20.1)	7.4 (16.5)	7.2 (16.5)	4.8 (10.7)	3.6 (8)

The application of a metaflumizone and amitraz spot-on formulation at 14 days intervals for 3 months was highly effective for the treatment of generalized demodectic mange and contributed to clinical recovery. Treatment with metaflumizone and amitraz spot-on formulation was shown to be easy to handle, safe and efficacious against canine demodectic mange. Currently, amitraz rinsing is the only treatment approved by the FDA for canine demodicosis, although its use can be very labour-intensive and may lead to toxic effects [6]. Previous studies evaluated the efficacy of amitraz rinse against juvenile- and adult-onset generalized demodicosis, demonstrating remission of the clinical signs after long-term amitraz therapy in 65% of the animals, of which 9% maintained the clinical remission with ongoing treatment [10]. Conversely, 25% of the animals did not respond to the therapy [10]. Similar remission rates have been reported for long-term, high-dose, daily therapy with ivermectin or milbemycin oxime and moxidectin [10,17]. However, treatment regimes with these macrocyclic lactones are costly, intensive (daily treatment), long (usually 3 months), and may be associated with adverse events [6].

Interestingly, treatment by metaflumizone plus amitraz spot-on was also effective in reducing the number of *M. pachydermatis *yeasts in each sampling site at +28 days p.t. Although *M. pachydermatis *cells could still be detected in the external ear canal, the population size decreased significantly from +28 days p.t. and at day +70, the yeast load was considered normal (i.e. about 20 CFU) [4].

These findings suggest that the alterations in the cutaneous microenvironment and/or in host defence mechanisms in canine skin may have influenced the mite development, *M. pachydermatis *and bacterial overgrowth thus leading to the appearance of skin lesions. However, while the acaricidal and antibiotic treatment may account for the reduction in mite number and presence of bacterial lesions, the reduction of *M. pachydermatis *needs further investigation. *M. pachydermatis *is an opportunistic pathogen and the metaflumizone and amitraz spot-on formulation, as well as antibiotic treatment, may have reduced yeast population as an additional effect of the acaricidal activity leading to the improvement of skin lesions.

In conclusion, the results of the case report presented here show that metaflumizone plus amitraz in a spot-on formulation, associated with antibiotic therapy, was highly effective for the treatment of generalized demodectic mange and this could contribute to the control of also these opportunistic infections. Nonetheless, the complex interactions occurring among the host immune response and the bacterial and yeast infections associated with canine demodectic generalized mange, need to be addressed in order to standardize effective and safe treatment with reduced administration times.

## Competing interests

The authors declare that they have no competing interests.

## Authors' contributions

VDT, and DO conceive the paper and wrote the manuscript. DO helped in the data interpretation. RPL, VDT, MS, CC, DO performed clinical examination and laboratory analyses.

## Supplementary Material

Additional file 1 **Table S1**. Number of *Demodex canis *adults, nymphs, larvae and eggs from skin scraping from anterior left leg (L), thorax (Tx) and tight (Ti) at different sampling times. Click here for file
