# Supplementary material for: Efficacy of Amitraz plus Metaflumizone for the treatment of canine demodicosis associated with Malassezia pachydermatis
Source: Parasit Vectors. 2009 Mar 5;2:13. doi: 10.1186/1756-3305-2-13 (PMC2654875; doi:10.1186/1756-3305-2-13)
Supplement: Additional file 1 — Table S1. Number of Demodex canis adults, nymphs, larvae and eggs from skin scraping from anterior left leg (L), thorax (Tx) and tight (Ti) at different sampling times. [file 1756-3305-2-13-S1.doc]

|  | 0 | | | | +14 | | | | +28 | | | | +42 | | | | +56 | | | | +70 | | | | +84 | | | |
| --- | --- | --- | --- | --- | --- | --- | --- | --- | --- | --- | --- | --- | --- | --- | --- | --- | --- | --- | --- | --- | --- | --- | --- | --- | --- | --- | --- | --- |
|  | L | Tx | Ti | Means (sd) | L | Tx | Ti | Means (sd) | L | Tx | Ti | Means (sd) | L | Tx | Ti | Means (sd) | L | Tx | Ti | Means (sd) | L | Tx | Ti | Means (sd) | L | Tx | Ti | Means (sd) |
| Eggs | 90 | 120 | 115 | 108.33 (16.0)^a^ | 128 | 102 | 100 | 110 (15.6)^b^ | 2 | 3 | 11 | 5.3 (4.9)^ab^ | - | - | - | 0 ^a b^ | - | - | - | 0 ^a b^ | - | - | - | 0 ^a b^ | - | - | - | 0 ^a b^ |
| Larvae | 47 | 60 | 48 | 51.7 (7.2)^c^ | 46 | 44 | 40 | 43.3  (3.1)^d^ | - | - | - | 0 ^cd^ | - | - | - | 0 ^cd^ | - | - | - | 0 ^cd^ | - | - | - | 0 ^cd^ | - | - | - | 0 ^cd^ |
| Nymphes | 13 | 8 | 6 | 9  (3.6)^e^ | 42 | 48 | 36 | 42  (6)^e^ | - | - | - | 0 ^e^ | - | - | - | 0 ^e^ | - | - | - | 0 ^e^ | - | - | - | 0 ^e^ | - | - | - | 0 ^e^ |
| Adults | 412 | 420 | 460 | 430.7 (25.7)^f^ | 277 | 250 | 220 | 249 (28.5)^f^ | 22 | 19 | 34 | 25  (7.9) ^f^ | 1 | 4 | 6 | 3.6 (2.5) ^f^ | - | - | 2 | 0.67  (1.1) ^f^ | - | - | - | 0 ^f^ | - | - | - | 0 ^f^ |

^a-f^ : Chi-square test : statistically significant differences (P< 0,05) were marked with the same letters.
